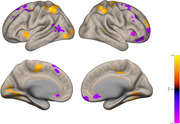# Neural correlates of emotion recognition in illiterate adults

**DOI:** 10.1002/alz.087562

**Published:** 2025-01-03

**Authors:** Vinicius Tinarelli Silva, João Victor de Faria Rocha, Clarisse Vasconcelos Friedlaender, Augusto Antunes, Lea T. Grinberg, Francisca Izabel Pereira Maciel, Paulo Caramelli, Elisa de Paula França Resende

**Affiliations:** ^1^ Universidade Federal de Minas Gerais, Belo Horizonte, Minas Gerais Brazil; ^2^ Alliar Medicina Diagnóstica, Belo Horizonte, Minas Gerais Brazil; ^3^ University of California, San Francisco, San Francisco, CA USA

## Abstract

**Background:**

The salience network (SN) functions as a dynamic switch between the default mode network (DMN) and the frontoparietal network (FPN), aligning with salience and cognitive demand. Dysfunctions in SN activity within the cognitive and affective domains are linked to a wide range of deficits and maladaptive behavioral patterns in various clinical disorders. Emotion recognition is pivotal in social interactions and can be affected in neurodegenerative disorders. The neural correlates of emotion recognition are well studied in adults with high levels of education, but few studies exist on neural correlates of emotion cognition in illiterate individuals.

**Method:**

Cognitively healthy illiterate adults (with barely no formal education) underwent neuropsychological evaluation and brain MRI scans. Emotion recognition was assessed using the Ekman test. The CONN toolbox was used for functional brain connectivity analysis. We then correlated the Ekman total score with the salience network connectivity using a voxel threshold of p < 0.05 and a cluster threshold of p < 0.05.

**Result:**

A total of 108 participants aged 57.7 (±9.51) years, with 2.0 (±2.3) years of education, of which 72.6% were women were recruited, eight participants declined the MRI scan and five participants were excluded because of structural lesions found in the MRI leaving out 95 participants. Ekman total score was 19.9 ±4.3. There was a significant correlation between the Ekman total score and regions in the salience network, with the most prominent voxels at the Superior Parietal Lobule Left (407 voxels covering 28%); Frontal Pole Right (370 voxels covering 5%) and Cerebellum Crus2 Left (221 voxels covering 11%).

**Conclusion:**

We can see a correlation between the clusters of the salience network and the total score on the Eckman test in illiterate individuals. This suggests that the salience network is also related to social cognition in this particular group.